# The Impact of Genetic Polymorphisms on Anterior Cruciate Ligament Injuries in Athletes: A Meta-Analytical Approach

**DOI:** 10.3390/biology12121526

**Published:** 2023-12-15

**Authors:** Alpay Bulbul, Erdal Ari, Necdet Apaydin, Gokhan Ipekoglu

**Affiliations:** 1 Department of Physical Education and Sports, Faculty of Sport Sciences, Adnan Menderes University, Aydın 09000, Turkey; abulbul60@adu.edu.tr; 2 Department of Physical Education and Sports, Faculty of Sport Sciences, Ordu University, Ordu 52200, Turkey; erdalari@odu.edu.tr (E.A.); necdetapaydin@odu.edu.tr (N.A.)

**Keywords:** COL1A1, COL3A1, COL5A1, COL12A1, ACL, athletes

## Abstract

**Simple Summary:**

This investigation delves into the association between genetic variations in collagen-related genes (specifically, COL1A1, COL3A1, COL5A1, and COL12A1) and the predisposition of athletes to anterior cruciate ligament (ACL) injuries. Through a meticulous meta-analysis of 19 case–control studies encompassing 9921 individuals, a noteworthy association was found between the presence of the G allele in the COL1A1 gene (rs1107946) and a reduced risk of ACL injuries. Contrarily, the A allele in the COL12A1 gene (rs970547) exhibited an association with an elevated susceptibility to ACL injuries. Moreover, no statistically significant associations were observed for COL3A1 (rs1800255) and COL5A1 (rs12722) gene variants. These findings provide valuable insights into the genetic factors influencing ACL injuries in athletes, contributing to the potential development of targeted preventive measures and enhancing our understanding of the genetic aspects of sports-related ligament injuries.

**Abstract:**

This meta-analysis aimed to investigate the association between genetic polymorphisms in Collagen type 1 alpha-1 (COL1A1), Collagen type 3 alpha-1 (COL3A1), Collagen type 5 alpha-1 (COL5A1), and Collagen type 12 alpha-1 (COL12A1) genes and anterior cruciate ligament (ACL) injuries in athletes. A systematic search was diligently conducted on the PubMed and Web of Science databases to identify relevant studies on 5–9 September 2023. Only case–control studies were included in the meta-analysis. A total of 19 studies were reviewed, involving the analysis of 3522 cases and 6399 control subjects. Data relevant to the study objectives were extracted from these chosen studies and subsequently analyzed using either a random-effects or fixed-effects model. It indicates that individuals carrying the G allele in the COL1A1 (rs1107946) gene have a decreased risk of anterior cruciate ligament injuries (OR: −0.27, 95% CI: −0.42 to −0.12, *p* < 0.001). A similar relationship was observed in the dominant model, but this relationship was reversed in the recessive model (OR: 0.69, 95% CI: 0.33 to 1.05, *p* < 0.001). However, no significant associations were found in the COL3A1 (rs1800255) and COL5A1 (rs12722) genes. In the COL12A1 (rs970547) gene, the A allele was associated with an increased risk of anterior cruciate ligament injuries (OR: 0.18, 95% CI: 0.01 to 0.36, *p* = 0.041). This meta-analysis suggests that genetic variants in COL1A1 (rs1107946) and COL12A1 (rs970547) may be associated with ACL injuries in athletes. However, COL3A1 rs1800255 and COL5A1 rs12722 gene variants do not appear to have a significant association with these injuries.

## 1. Introduction

Anterior cruciate ligament (ACL) ruptures are one of the most common and serious sports injuries. They can lead to both acute functional impairments and chronic early de-generative joint conditions [1]. Several internal and external factors are known to influence sports injuries, including the characteristic features of different sports disciplines. Actions involving changes in direction, which are frequently performed in many sports, have been reported to be associated with ACL ruptures [2]. ACL ruptures can occur during actions involving changes in direction even without any collision, contest, or contact within a competition [3]. The characteristic features of sports disciplines, athletes’ physical fitness levels, field conditions, impact, and trauma are recognized as external factors that can affect ACL ruptures.

In addition to external factors, there are internal factors that influence ACL ruptures. Among internal factors, genetic factors are considered the most significant risk factors for ACL ruptures [4]. Within the scope of genetic factors, genes involved in the structure of type 1 collagen are particularly prominent. Type 1 collagen is a fibril-forming collagen that is highly present in bones, tendons, and ligaments [3]. Type 1 collagen is formed by two α1 chains encoded by the COL1A1 (chr17q21.33) gene and one α2 chain encoded by the COL1A2 (chr7q21.3) gene [3,5]. The rs1107946 variant of the COL1A1 gene has been reported to be associated with the risk of acute soft tissue injuries related to sports, including shoulder dislocations, cruciate ligament ruptures, and Achilles tendon ruptures [6,7,8,9,10,11]. Mutations occurring in the COL1A1 gene have been reported to be associated with anterior cruciate ligament injuries. Specifically, mutations in the COL1A1 gene are implicated in osteogenesis imperfecta, a condition characterized by brittle collagen structures that lead to multiple bone fractures, including ligaments, tendons, and teeth [5,12]. These mutations increase the fragility of collagen tissues that make up the structure of ligaments and tendons, posing a risk factor for anterior cruciate ligament injuries. Similarly, the α1 chains of type 5 and type 12 collagen, encoded by the COL5A1 and COL12A1 genes, respectively, also play a role in ligaments’ and tendons’ structure. Approximately 75% of the dry weight of the solid components constituting ligaments consists of collagen, with approximately 85% of this being type 1 collagen [13]. The most important structural components of ligaments are particularly type 1 and type 5 collagens [14]. COL1A1 gene, which is associated with type 1 collagen, is linked to cruciate ligament ruptures, while COL5A1 gene, which is associated with type 5 collagen, is linked to Achilles tendon injuries [12].

COL5A1 gene, on the other hand, encodes another important collagen, type 5 collagen, found in the structure of ligaments and tendons. Type 5 collagen is necessary to increase the durability of ligaments and provide flexibility to tendons. COL5A1 gene directs the production of type 5 collagen, which can affect the strength of athletes’ ligaments and tendons. In this context, the rs12722 variant of the COL5A1 gene is being investigated for its association with acute soft tissue injuries related to sports, especially ACL ruptures [14,15,16].

Another collagen found in the structure of ligaments is type 3 collagen. Type 3 colla-gen, a homotrimer formed by three α1 chains, is a product of the COL3A1 gene [17]. Type 3 collagen, encoded by COL3A1, regulates the strength and flexibility of the tissues in which it is found and functionally adapts during the body’s response to injuries. Furthermore, the rs1800255 polymorphism of the COL3A1 gene has been shown to be associated with ACL ruptures [18].

COL12A1 gene is a member of the collagen family and encodes type 12 collagen, which is one of the structural components of ligaments. Type 12 collagen, responsible for regulating the durability and flexibility of ligaments, plays an important role in protecting the body’s tissues from injuries. COL12A1 gene codes for the essential components of ligaments required to maintain their structural integrity and stability [19]. In this context, the rs970547 variant of the COL12A1 gene may be associated with ACL ruptures and other acute soft tissue injuries related to sports [20]. However, the role of this gene and the effect of this variant are not yet fully understood. This study will examine the potential effect of the rs970547 variant of the COL12A1 gene on ACL ruptures in more detail.

The COL1A1, COL3A1, COL5A1, and COL12A1 genes, which encode the collagen molecules that constitute ligaments, have different single-nucleotide variants. Among these variants, the rs1107946 of COL1A1, rs1800255 of COL3A1, rs12722 of COL5A1, and rs970547 polymorphisms of COL12A1 have been the subject of numerous studies investigating their association with ACL ruptures [11,15,19,20,21]. There is no consistency among the findings of these studies. Moreover, there is no existing meta-analysis study in the literature that evaluates the association between the COL1A1, COL3A1, COL5A1, and COL12A1 genes and anterior cruciate ligament injuries in athletes with a case–control focus. Therefore, it is believed that this meta-analysis study will contribute to the literature. In this context, the aim of the study is to investigate the relationship between the variants of COL1A1 (rs1107946), COL3A1 (rs1800255), COL5A1 (rs12722), and COL12A1 (rs970547) genes and ACL ruptures through meta-analysis.

## 2. Materials and Methods

### 2.1. Data Collection and Selection of Relevant Studies

This study commenced with a systematic search in the PubMed and Web of Science databases. Keywords such as “COL1A1 polymorphism”, “COL3A1 polymorphism”, “COL5A1 polymorphism”, “COL12A1 polymorphism”, and “genetic polymorphism” were used in combination with the term “anterior cruciate ligament” or “ACL”. To identify relevant studies, the titles, abstracts, and full texts of the research were reviewed.

**Figure 1:**
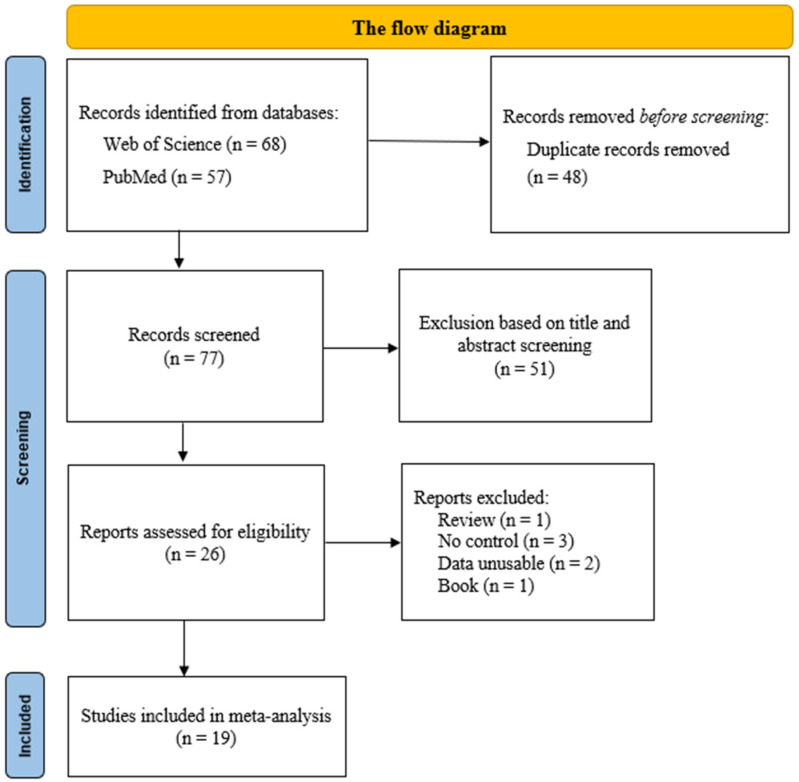
The flow diagram of included/excluded studies in the meta-analysis.

As a result of data collection, a total of 125 studies were identified, of which 19 met the inclusion criteria for meta-analysis (Figure 1). The inclusion criteria are as follows: (1) it must be an original study; (2) the study examines the relationship between COL1A1, COL3A1, COL5A1, and COL12A1 gene polymorphism and anterior cruciate ligament (ACL) injury; (3) the study includes both a case and a control group; (4) the study provides sufficient data to calculate an odds ratio or risk ratio, along with a 95% confidence interval for these data; (5) the study is written in English; and (6) the participants in the control group should consist of healthy individuals who have not experienced any ligament injuries. The exclusion criteria are as follows: (1) the study is a review article; (2) the study does not include a control group; (3) the study is not relevant to the research question; (4) the study’s full texts are not accessible or necessary data are not provided; and (5) the control group participants have experienced at least one type of ligament injury. While conducting a literature review, the genders of the sample groups were not a restrictive parameter. After determining the studies to be included, due to the low number of studies with female participants (<3), data from female participants were not included in the meta-analysis.

### 2.2. Data Extraction and Evaluation

The selection of relevant studies and data extraction were carried out independently by two independent review experts. Information extracted from each study included the basic characteristics of the study (publication year, country, study design), population characteristics (age, gender, ethnic group), and information about the gene variants studied (COL1A1, COL3A1, COL5A1, COL12A1).

### 2.3. Study Quality Assessment

Publication bias was assessed using funnel plots and statistical tests (e.g., Egger’s test) to detect potential bias in the literature search. Risk of bias within individual studies was assessed using established tools such as the Newcastle–Ottawa Scale for case–control and cohort studies or the Cochrane Risk of Bias tool for randomized controlled trials.

### 2.4. Statistical Analysis

Statistical procedures for the analysis of the study’s data were conducted using Jamovi software (version 2.3). These analyses were performed to assess the impact of COL1A1, COL3A1, COL5A1, and COL12A1 gene variants on anterior cruciate ligament injuries. Statistical results were presented with odds ratios (ORs) and 95% confidence intervals (CIs). Cochran’s Q statistic and I² statistic were employed to assess heterogeneity. In the presence of heterogeneity, a random-effects model was preferred. A significance level of *p* < 0.05 was considered for statistical analyses.

## 3. Results

In the context of this meta-analysis, firstly, keywords were identified. Then, 125 articles published in journals scanned in the Web of Science and PubMed databases were detected. After examining the titles, abstracts, and full texts of the identified articles, studies that did not meet the inclusion criteria were excluded. After filtering, 19 articles were included in this meta-analysis. While analyzing the articles obtained from the databases, studies that were not categorized as ACL (including other ligament injuries), studies without a control group, and studies with unclear allele and genotype data were not included in the analysis.

In Table 1, descriptive data for all the studies included in the meta-analysis are presented. This information includes the study’s lead author, the year of the study, the race of the individuals comprising the sample group, genetic and polymorphism information, the number of individuals in the ACL and control groups, and the Newcastle–Ottawa scale values.

### 3.1. The COL1A1 (rs1107946) Allele and Genotype Distribution (ACL vs. Control)

**Figure 2:**
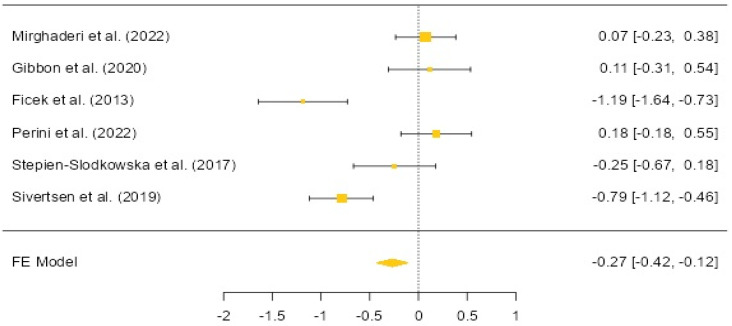
COL1A1 rs1107946 allele-based OR (95%CI) and forest plot [3,6,7,9,11,21].

Six articles were identified that analyzed individuals with a history of ACL injury and healthy individuals in the control group for the COL1A1 rs1107946 polymorphism [3,6,7,9,11,21] (Figure 2). In these six studies, a total of 891 athletes with ACL injuries and 1675 participants in the control group were analyzed (Table 2). According to genetic analysis, individuals carrying the G allele were significantly associated with a reduced risk of anterior cruciate ligament injury (OR: −0.27, 95% CI: −0.42 to −0.12, *p* < 0.001). In the dominant model, individuals carrying the G allele also exhibited a significantly lower risk of anterior cruciate ligament injury (OR: −0.20, 95% CI: −0.38 to −0.01, *p* = 0.034). Conversely, in the recessive model, individuals carrying the G allele were found to have a significantly increased risk of anterior cruciate ligament injury (OR: 0.69, 95% CI: 0.33 to 1.05, *p* < 0.001). High heterogeneity was observed in both allele-based and dominant model-based results (87.22% and 87.34%, respectively), whereas there was no heterogeneity in the random-effects model-based results (0%). Egger’s test results did not indicate publication bias in allele-based and dominant model-based results (*p* = 0.094 and *p* = 0.001).

### 3.2. The COL3A1 (rs1800255) Allele and Genotype Distribution (ACL vs. Control)

**Figure 3:**
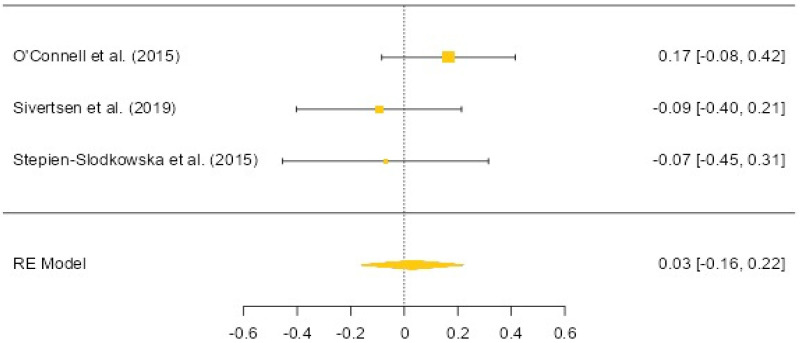
COL3A1 rs1800255 allele-based OR (95%CI) and forest plot [11,16,19].

In three studies where the ACL group and the control group were analyzed for the COL3A1 rs1800255 polymorphism, a total of 568 athletes with a history of ACL injury and 1271 controls were examined [11,16,19] (Figure 3). There was no significant association found between anterior cruciate ligament injury and individuals carrying the G allele (OR: 0.03, 95% CI: −0.16 to 0.22, *p* = 0.756). In the dominant model, there was also no significant relationship between anterior cruciate ligament injury and individuals carrying the G allele (OR: 0.11, 95% CI: −0.15 to 0.37, *p* = 0.402). Both results exhibited low heterogeneity (14.36% and 27.61%, respectively). Egger’s test results did not indicate publication bias (allele-based *p* = 0.245, DM-based *p* = 0.951).

### 3.3. The COL5A1 (rs12722) Allele and Genotype Distribution (ACL vs. Control)

**Figure 4:**
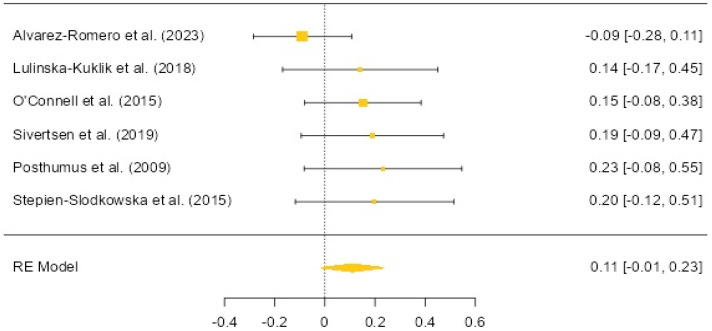
COL5A1 rs12722 allele-based OR (95%CI) and forest plot [11,14,15,16,18,22].

In the meta-analysis examining the relationship between the COL5A1 rs12722 polymorphism and anterior cruciate ligament (ACL) injuries, a total of 1413 athletes with a history of ACL injury and 2006 controls were analyzed across six studies [11,14,15,16,18,22] (Figure 4). According to the study’s results, there was no significant association found between anterior cruciate ligament injury and individuals carrying the T allele (OR: 0.11, 95% CI: 0.01 to 0.23, *p* = 0.081). In the dominant model, there was also no significant relationship between anterior cruciate ligament injury and individuals carrying the T allele (OR: 0.14, 95% CI: −0.02 to 0.31, *p* = 0.088). Both results exhibited low heterogeneity (22.59% and 4.84%, respectively). Egger’s test results indicated publication bias for DM-based results (*p* = 0.050), but not for allele-based results (*p* = 0.384).

### 3.4. The COL12A1 (rs970547) Allele and Genotype Distribution (ACL vs. Control)

**Figure 5:**
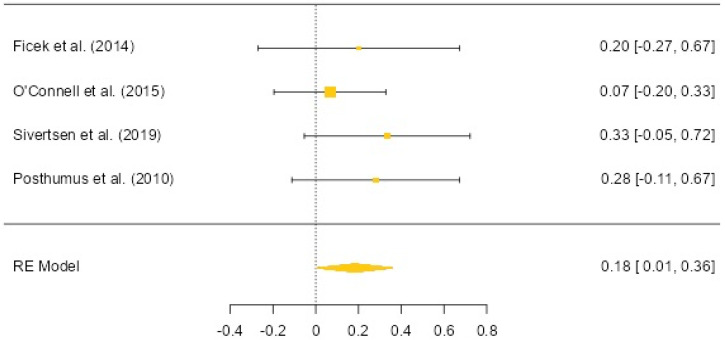
COL12A1 rs970547 allele-based OR (95%CI) and forest plot [11,15,20,23].

The meta-analysis included four articles that examined the relationship between the COL12A1 rs970547 polymorphism and ACL injuries [11,15,20,23] (Figure 5). In these included studies, a total of 650 athletes with a history of ACL injury and 1447 controls were examined. The results have shown that the A allele frequency was significantly higher in athletes with anterior cruciate ligament injuries (OR: 0.18, 95% CI: 0.01 to 0.36, *p* = 0.041). In the dominant model, individuals carrying the A allele were found to have a significant association with anterior cruciate ligament injuries (OR: 0.19, 95% CI: −0.01 to 0.39, *p* = 0.062). However, in the recessive model, there was no significant relationship between carrying the A allele and anterior cruciate ligament injuries (OR: 0.19, 95% CI: −0.28 to 0.65, *p* = 0.432). Heterogeneity was low in both allele-based and DM-based results (0%), and there was no heterogeneity in RM-based results (0%). Egger’s test results did not suggest publication bias (allele-based *p* = 0.321, DM-based *p* = 0.707).

## 4. Discussion

**Figure 6:**
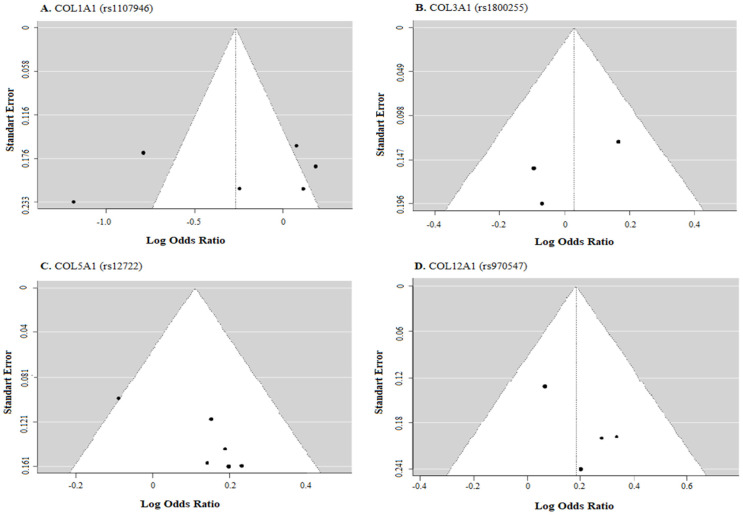
Allele-based funnel plots for (**A**) COL1A1 (rs1107946), (**B**) COL3A1 (rs1800255), (**C**) COL5A1 (rs12722), and (**D**) COL12A1 rs970547 polymorphisms.

In this study, the findings of our research investigating the relationship between COL1A1 (rs1107946), COL3A1 (rs1800255), COL5A1 (rs12722), and COL12A1 (rs970547) genes and anterior cruciate ligament (ACL) ruptures were examined and analyzed (Figure 6). Nineteen studies were included in this analysis, encompassing a total of 3522 cases and 6399 control group subjects.

The analysis of data from the reviewed studies revealed significant differences in the COL1A1 (rs1107946) gene in terms of major, dominant, and recessive genetic models when comparing ACL injuries to the control group (*p* < 0.05). Specifically, individuals with the G major allele in the COL1A1 (rs1107946) gene were found to have fewer ACL injuries. When examining genotypes within the COL1A1 (rs1107946) gene, ACL injuries were less frequent in the dominant model, but more frequent in the recessive model. It is important to note that the findings regarding the relationship between COL1A1 (rs1107946) and ACL injuries vary in the literature.

Mirghaderi et al. [3] reported that the frequency of the G allele was higher in a group of athletes with ACL injuries compared to the control group, but this difference was not statistically significant. Similarly, they found no significant differences in ACL injuries when considering codominant, dominant, or recessive genotypes. Gibbon et al. [6] also concluded that there were no significant differences in the genotypes and allele frequency distribution of COL1A1 (rs1107946) between control groups and those with Achilles tendonitis and ACL rupture. The findings of this study contrast with those of previous re-search, as Ficek et al. [7] found no significant differences in allele frequency and genotype distribution of COL1A1 (rs1107946) between groups with ACL ruptures and control groups. Additionally, a study conducted on athletes found no significant differences in allele and genotype distribution of COL1A1 rs1107946 in individuals with ACL tears compared to those without [22]. Stepien-Slodkowska et al. [9] reported no significant differences in allele and genotype (dominant and recessive) distribution of COL1A1 (rs1107946) in recreational skiers with ACL ruptures compared to a control group without any ligament or tendon injuries. In a study by Sivertsen et al. [11], the minor allele and genotype distribution of COL1A1 rs1107946 did not significantly differ between Finnish and Norwegian elite female athletes with and without ACL injuries.

In summary, while the findings of these studies suggest that COL1A1 (rs1107946) may not be significantly associated with ACL injuries, the results of the current meta-analysis indicate significant differences in allele and genotype distribution based on the occurrence of ACL injuries.

The analysis revealed no significant differences in allele and genotype distribution of COL3A1 (rs1800255) between ACL injury and control groups. This result is consistent with the findings of O’Connell et al. [15], who reported no significant differences in allele and genotype distribution of COL3A1 rs1800255 in South African individuals with ACL injuries compared to control groups. However, in the same study, among Polish individuals, it was found that the AA genotype was higher in the ACL injury group compared to the control group, while the allele distribution did not differ significantly. Similar to this research, Sivertsen et al. [11] found no significant differences in the allele distribution of COL3A1 rs1800255 between Norwegian and Finnish elite female athletes with and with-out ACL injuries. In a study by Ipekoglu et al. [24] on Turkish male professional soccer players, there were no significant differences in allele and genotype distribution of COL3A1 rs1800255 based on the occurrence of ACL injuries. However, it is worth noting that in recreational skiers, significant differences were reported in the recessive model, where ACL ruptures were more frequent [18].

These findings suggest that COL3A1 (rs1800255) may not be significantly associated with ACL injuries, although there may be variations based on ethnicity or sport type. Further meta-analyses considering gender-specific data could provide a more comprehensive understanding of the relationship between COL3A1 (rs1800255) and ACL injuries.

The analysis of COL5A1 (rs12722) showed no significant differences in allele and genotype distribution between ACL injury and control groups. These findings are in line with previous research. Lyu et al. [25] investigated the relationship between the COL5A1 (rs12722) polymorphism and ACL injuries in a meta-analytic study, and their findings align with the results obtained in this study. In both studies, no statistically significant differences were observed in both the dominant (OR: 0.75, 95% CI: 0.59 to 0.95, *p* = 0.96) and recessive models (OR: 0.64, 95% CI: 0.48 to 0.86, *p* = 0.245). It is believed that the difference in odds ratios (ORs) may stem from the inclusion of different studies. While this study includes ACL groups consisting exclusively of athletes, the other meta-analysis has included ACL groups comprising both athletes and sedentary individuals in their analysis. Alvarez-Romero et al. [22] found no significant differences in the genotype and allele distribution of COL5A1 rs12722 among different South African, Australian, and Japanese participants with and without ACL injuries. Similarly, Lulinska-Kuklik et al. [16] reported that the genotype and allele distribution of COL5A1 rs12722 did not significantly differ between soccer players who had experienced ACL injuries and those who had not. In the study by Sivertsen et al. [11], there were no significant differences in allele distribution of COL5A1 rs12722 between Norwegian and Finnish elite female athletes with and without ACL injuries. Posthumus et al. [14] also found no significant differences in allele and genotype distribution of COL5A1 rs12722 among South African individuals with ACL injuries compared to control groups. Similarly, Stepien-Slodkowska et al. [8] found no significant differences in allele and genotype distribution of COL5A1 rs12722 in male recreational skiers based on ACL injury status. O’Connell et al. [15] reported only a higher frequency of the TT and TC genotypes of COL5A1 rs12722 in South African women with ACL injuries compared to those without injuries. However, in Polish women and South African men and women, there were no significant differences in genotype distribution based on injury status.

These results suggest that COL5A1 (rs12722) may not be significantly associated with ACL injuries, and the relationship appears consistent across different populations and sports. However, additional studies focusing on COL5A1 (rs12722) polymorphism and ACL injuries may contribute to a more comprehensive assessment.

The research findings reveal a significant difference in the allele distribution of COL12A1 rs970547 polymorphism between the anterior cruciate ligament injury group and the control group, whereas no significant difference was observed in genotype distribution between the groups. Generally, in research related to COL12A1, there is no significant difference in allele and genotype distribution between anterior cruciate ligament in-jury and control groups. Lv et al. [26] found no statistically significant difference in the allele model of the meta-analysis examining the relationship between COL12A1 rs970547 polymorphism and ACL injuries (OR: 0.91, 95% CI 0.77, 1.08; *p* = 0.28). This result contradicts the findings in our study. It is speculated that the difference may arise from the inclusion of both athlete and sedentary ACL groups in the other study, while our study only includes ACL groups consisting exclusively of athletes. The meta-analysis conducted by Lyu et al. [25] demonstrates a similarity with this study in both dominant (OR: 0.74, 95% CI 0.59, 0.93; *p* = 0.206) and recessive model (OR: 1.15, 95% CI 0.72, 1.83; *p* = 0.408) genotype analyses. Ficek et al. [23] demonstrated that there was no significant difference in genotype and allele distribution among male soccer players concerning anterior cruciate ligament injury status. Similarly, Sivertsen et al. [11] found no significant difference in genotype and allele distribution of COL12A1 rs970547 polymorphism among Norwegian and Finnish female athletes participating in team sports, based on anterior cruciate ligament injury status. In a study by Posthumus et al. [20] conducted on Caucasian male and female individuals with anterior cruciate ligament injuries, it was reported that there was no significant difference in genotype distribution compared to non-injured individuals, although a significant difference in genotype distribution was observed among female individuals with anterior cruciate ligament injuries compared to those without. Specifically, female individuals with anterior cruciate ligament injuries had a higher prevalence of the AA genotype. O’Connell et al. [15] also indicated that there was no significant difference in the genotype distribution of COL12A1 rs970547 polymorphism among Caucasian (South African and Polish) male individuals based on anterior cruciate ligament injury status. In the same study, it was reported that there was no significant difference in genotype distribution among South African female individuals based on anterior cruciate ligament injury status, whereas among Polish female individuals with anterior cruciate ligament injuries, the AA genotype was significantly more prevalent. Notably, the findings of the studies conducted by O’Connell et al. [15] and Posthumus et al. [20] suggest that significant differences in the genotype distribution of COL12A1 rs970547 polymorphism may be specific to female individuals. However, allele distribution in this study showed a significant difference based on anterior cruciate ligament injury status, while genotype distribution did not differ significantly between groups. The genotype distribution findings of COL12A1 rs970547 polymorphism in this study are in partial agreement with some literature findings. When considering studies in the literature examining the genotype distribution of COL12A1 rs970547 polymorphism in relation to anterior cruciate ligament injury, the notable difference in findings highlights the need for further comprehensive research. Therefore, it is suggested that additional studies focusing on allele distribution are necessary for a more comprehensive evaluation in this regard.

The most significant limitation of this study is the insufficient size of the sample groups in the studies included in the meta-analysis. Data limitations arise from a scarcity of studies meeting the inclusion criteria in the literature, an inadequate representation of female participants in the sample groups of included studies, and a lack of a sufficient data pool for interpreting races based on certain genotypes. These are among the other limitations of this study.

## 5. Conclusions

In conclusion, this meta-analysis suggests that COL1A1 (rs1107946) and COL12A1 (rs970547) gene variants may be associated with anterior cruciate ligament injuries, while COL3A1 rs1800255 and COL5A1 rs12722 gene variants do not show a significant relationship with these injuries. These results particularly differ from allele distribution findings reported in other studies in the literature. This underscores the complexity of genetic factors in anterior cruciate ligament injury risk and emphasizes the need for further research to clarify this relationship. This research highlights the importance of considering genetic factors to better understand the risk of injuries among athletes and to develop prevention strategies.

## Figures and Tables

**Table 1:** Descriptive information of the studies included in the meta-analysis.

Study	Race	Name Genes (rs Number)	Total Athletes	Total Controls	Newcastle–Ottawa Scale			
					Sel	Com	Exp	Total
Mirghaderi et al. (2022) [22]	C	COL1A1 (rs1107946)	200	200	3	2	3	8
Gibbon et al. (2020) [6]	A	COL1A1 (rs1107946)	197	225	3	1	3	7
Ficek et al. (2013) [7]	E	COL1A1 (rs1107946)	91	143	3	1	3	7
Perini et al. (2022) [21]	M	COL1A1 (rs1107946)	146	192	3	2	3	8
Stepien-Slodkowska et al. (2017) [9]	E	COL1A1 (rs1107946)	138	183	3	1	2	6
Sivertsen et al. (2018) [11]	E	COL1A1 (rs1107946)	119	732	2	2	2	6
O’Connell et al. (2015) [15]	C	COL3A1 (rs1800255)	311	356	3	2	3	8
Sivertsen et al. (2018) [11]	E	COL3A1 (rs1800255)	119	732	2	2	2	6
Stepien-Slodkowska et al. (2015) [8]	E	COL3A1 (rs1800255)	138	183	3	1	2	6
Alvarez-Romero et al. (2023) [22]	M	COL5A1 (rs12722)	582	308	2	2	2	6
Lulińska-Kuklik et al. (2018) [16]	E	COL5A1 (rs12722)	134	211	3	2	3	8
O’Connell et al. (2015) [15]	C	COL5A1 (rs12722)	311	356	3	2	3	8
Sivertsen et al. (2018) [11]	E	COL5A1 (rs12722)	119	732	2	2	2	6
Posthumus et al. (2009) [14]	A	COL5A1 (rs12722)	129	216	3	2	3	8
Stepien-Slodkowska et al. (2015) [8]	E	COL5A1 (rs12722)	138	183	3	1	2	6
Ficek et al. (2014) [23]	E	COL12A1 (rs970547)	91	143	3	1	3	7
O’Connell et al. (2015) [15]	C	COL12A1 (rs970547)	311	356	3	2	3	8
Sivertsen et al. (2018) [11]	E	COL12A1 (rs970547)	119	732	2	2	2	6
Posthumus et al. (2009) [14]	A	COL12A1 (rs970547)	129	216	3	2	3	8

A: Asian; C: Caucasian; E: European; M: Mix; Sel: Selection; Com: Comparability; Exp: Exposure.

**Table 2:** Overall analyses of the association between the allele-based OR of the investigated genes and ACL injury.

	Meta Analysis							Heterogeneity			Publ. Bias
	Gene	n	MA	OR	Lower CI	Upper CI	*p*	I^2^	Q (df)	*p*-Value of Q	Egger’s
**Allele-based OR (95%Cl)**	COL1A1 (rs1107946)	6	G	−0.27	−0.42	−0.12	0.001 *^F^	87.22%	39.135	0.001	0.094
	COL3A1 (rs1800255)	3	G	0.03	−0.16	0.22	0.756 ^R^	14.36%	2.018	0.365	0.245
	COL5A1 (rs12722)	6	T	0.11	−0.01	0.23	0.081 ^R^	22.59%	5.270	0.384	0.050
	COL12A1 (rs970547)	4	A	0.18	0.01	0.36	0.041 *^R^	0%	1.594	0.661	0.321
**DM-based OR (95%Cl)**	COL1A1 (rs1107946)	6	G	−0.20	−0.38	−0.01	0.034 *^F^	87.34%	39.497	0.001	0.001
	COL3A1 (rs1800255)	3	G	0.11	−0.15	0.37	0.402 ^R^	27.61%	2.726	0.256	0.951
	COL5A1 (rs12722)	6	T	0.14	−0.02	0.31	0.088 ^R^	4.84%	3.733	0.589	0.307
	COL12A1 (rs970547)	4	A	0.19	−0.01	0.39	0.062 ^R^	0%	1.768	0.622	0.707
**RM-based OR (95%Cl)**	COL1A1 (rs1107946)	6	G	0.69	0.33	1.05	0.001 *^F^	87.66%	40.512	0.001	0.023
	COL3A (rs1800255)	3	G	0.18	−0.26	0.62	0.417 ^F^	72.51%	7.275	0.026	0.012
	COL5A1 (rs12722)	6	T	−0.24	−0.48	0.01	0.059 ^R^	34.88%	6.901	0.228	0.302
	COL12A1 (rs970547)	4	A	0.19	−0.28	0.65	0.432 ^R^	0%	1.881	0.598	0.522

* *p* < 0.05, Abbreviation: MA, major allele; OR, odds ratio; CI, confidence interval; R, random effect; F, fixed effect.

## Data Availability

Individual participant data that underlie the results reported in this article can be obtained by contacting the corresponding author, gokhanipekoglu@odu.edu.tr.
